# Preserving Traditional Botanical Knowledge: The Importance of Phytogeographic and Ethnobotanical Inventory of Peruvian Dye Plants

**DOI:** 10.3390/plants6040063

**Published:** 2017-12-18

**Authors:** José Mostacero León, Segundo E. López Medina, Helmut Yabar, Jordan De La Cruz Castillo

**Affiliations:** 1 Faculty of Biological Sciences, National University of Trujillo, Jr. San Martin 392, Trujillo 13007, Peru; jmostacero@unitru.edu.pe (J.M.L.); slopezm@unitru.edu.pe (S.E.L.M.); jdelacruz@unitru.edu.pe (J.D.L.C.C.); 2 Graduate School of Life and Environmental Sciences, University of Tsukuba, 1-1-1 Tennodai, Tsukuba, Ibaraki 305-8587, Japan

**Keywords:** taxonomic inventory, botanical explorations, Herbarium Truxillense, endemic species, Andes Mountains

## Abstract

Peru is a megadiverse country with native species of all kinds, including dye plants, which have been used for hundreds of years by the local population. Despite the fact that many of these natural dyes are of a superior quality compared to synthetic ones and do not have the harmful effects that the latter may cause to human health, due to the lack of documentation and dissemination, ethnobotanical knowledge is unfortunately being lost with the passing of generations. In order to preserve and spread such valuable knowledge, this study conducted a comprehensive taxonomic, phytogeographic, and ethnobotanical inventory of dye plants based on periodical botanical explorations in selected locations of Northern Peru during the span of two decades. A critical review of the specialized bibliography was then carried out and the findings were verified with the personal knowledge and experience of both the researchers and the local and regional people. The results of the inventory record 32 species of dye plants from Northern Peru distributed in 22 families, of which the following stand out due to the number of species: Fabaceae (5), Anacardiaceae (2), Annonaceae (2), Asteraceae (2), Berberidaceae (2), Rosaceae (2), and Solanaceae (2). Of the 32 dye species identified, four are considered endemic from Peru: *Berberis buceronis* J.F. Macbr., *Caesalpinia paipai* Ruiz & Pav., *Coreopsis senaria* S.F. Blake & Sherf., and *Lomatia hirsuta* (Lam.) Diels. The study also found that species such as *Bixa orellana* L., *Indigofera suffruticosa* Mill., *Sambucus peruviana*, and the lichen *Usnea baileyi* (Stirton) Zahlbr have not been commercially exploited in Peru despite the fact that they already constitute a great economic source for several countries.

## 1. Introduction

Plant and animal domestication has been perhaps the most important development in the rise of human civilization [1]. Humans rely on plants for food, feed for their domestic animals, construction materials, medicines, paper, fibers, dyes, and cosmetics, etc. 

The use of dye plants and colorants from mineral and animal origin dates back to ancient times, and has been a very important resource for human beings [2]. Anthropological and archaeological studies and historians and chroniclers have analyzed the use of dyes through time, from the cave paintings of civilizations such as the Olmec, Maya, Aztec, Teotihuacan, Paracas, Tiawanako, and Chavín, as well as in diverse artistic representations in bicolor and polychrome pottery, painted murals, and in textile figures and fragments which demonstrate the use of paints and dyes not only in ceremonial objects, but also in fabric [3]. 

A megadiverse country like Peru has great botanical wealth [4,5] and is home to plants, which contain active coloring substances such as flavonoids, xanthones, quinones, and carotenoids [6,7], among other substances with high potential for use in the textile, cosmetic, and food industries. These have been cultivated and used until now by the local population, and in many regards, surpass artificial dyes. 

Natural dyes, such as those obtained from the *Bixa orellana* L., *Usnea baileyi* (Stirton) Zahlbr.b, *Alnus acuminata* H.B.K., *Berberis buceronis* Macb., *Bocconia integrifolia* Bonpl., *Caesalpinia spinosa* (Feuillée ex Molina) Kuntze, *Coreopsis senaria* Blake & Sherff., *Lomatia hirsuta* (Lam.) Diels, *Polylepis incan*a Kunth, *Hypericum laricifolium* Juss, and many other plants produce colors that are esthetically superior and environmentally friendly. Moreover, these natural dyes lack the substances that can cause injurious or allergic reactions in the consumers [8], produce attention deficit/hyperactivity in children [9,10], and/or have been prohibited by the Food and Drug Administration of the United States of America [11]. In addition, synthetic dyes generate water pollution, among other environmental effects [12]. Some studies have also demonstrated that fabrics colored with vegetable dyes fade less, or fade more harmonically and without jarring differences in color [13,14]. 

There have been various research initiatives related to ethnobotanical studies worldwide. Ivancheva and Stantcheva (2000) reported on the traditional use of medicinal plants in Bulgaria and identified 73 medicinal plants of 30 families [15]. Idolo et al. (2010) conducted an ethnobotanical inventory of one of the oldest national parks in Europe and highlighted the importance of nature preservation to preserve traditional knowledge [16]. Kaval et al. (2014) compiled ethnobotanical information on medicinal plants used in a Turkish region and found the extensive use of such plants for therapeutical purposes [17]. Amjad (2015) compiled the use of medicinal plants and other folk native plants of the Bana Valley in Pakistan to preserve the ethnobotanical knowledge of the region [18]. In the case of Peru, there have been some research initiatives on dye plants. Cornejo (1981), for instance, analyzed the dye plants in Huamanga province and their application in textile crafts [19]. Soukup (1986) compiled an inventory of the common names of Peruvian native plants including dye plants [20]. Rodriguez et al. (2017) analyzed data of Peruvian herbaria and identified some dye plants of La Libertad Region in Peru [21]. However, in all the cases, the studies only focused on the random inventory of the dye plants and some empirical applications. In this sense, the limited systematic documentation and dissemination of the traditional knowledge obtained through the empirical use of many plant species in Northern Peru is notorious. Because of this, establishing a link between the scientific and empirical knowledge of these plants is of the utmost importance [22,23,24]. This study attempts to fill this gap by not only providing a comprehensive inventory of the dye plants used in northern Peru, but also by compiling and disseminating their traditional uses and main characteristics based on the interviews conducted with local people. 

## 2. Material and Methods

**Figure 1:**
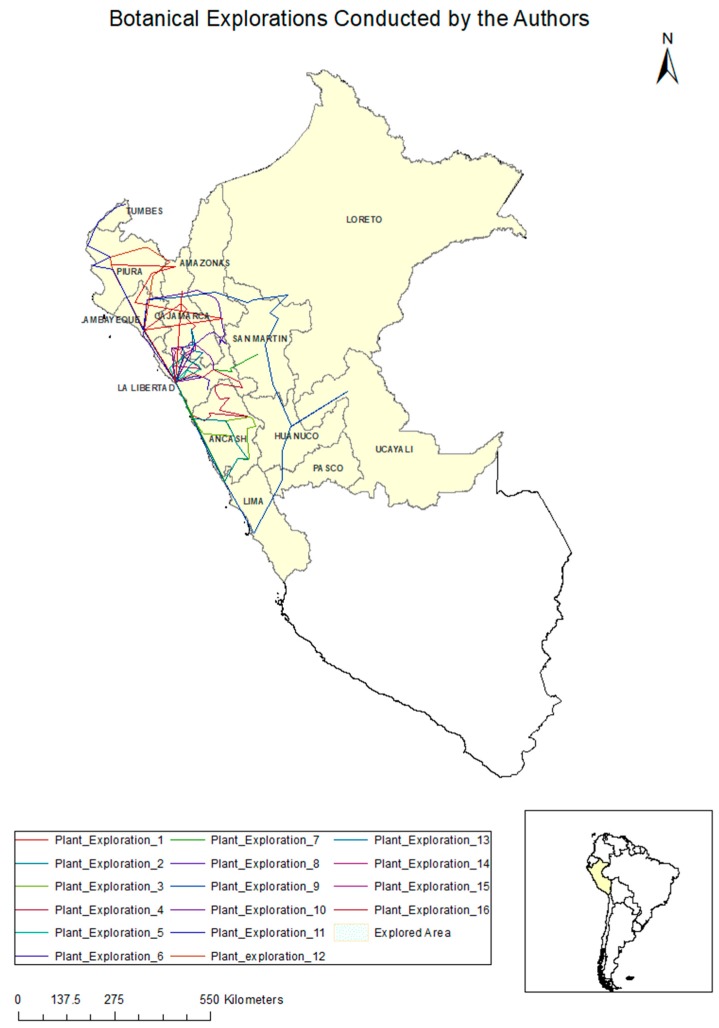
Botanical explorations conducted by the authors.

This study is based on the personal experience and observations of the authors. The authors carried out specimen collections during botanical excursions to various locations in the departments of Northern Peru. An average of two 10-day expeditions were carried out annually for two decades (from 1996 to 2016). A summary of the expedition routes is provided in Figure 1 and the details of the explored places are provided in Table 1.

This study is also based on the explorations, collections, and records of dye plant species used many generations ago available in the National University of Trujillo Herbarium also known as Herbarium Truxillense or HUT. These explorations gathered facts about the taxonomy, growth habit, altitudinal distribution, methods of propagation, parts of the plant utilized, and color produced from northern Peruvian dye plants. 

The information about common names, parts of the plants used, and the color produced by the species was obtained directly from surveys conducted with local populations, according to the labels accompanying the species registered in the HUT and notes recorded directly during the explorations.

The botanical specimens collected, once preserved, were registered in the HUT, and duplicates were sent to different herbaria, including the Missouri Botanical Garden, and the Chicago Field Museum in the United States, for taxonomic confirmation. It is worth mentioning that the specimens were identified using the taxonomic key referring to Peruvian flora in accordance with [25,26].

## 3. Results and Discussion

Scientific names, families, common names, growth habit, altitudinal distribution, types of plants propagation, parts of the plants used, and colors obtained from 32 species of dye plants collected in Northern Peru are given in Table 2. The 32 species were grouped into 29 genera and 22 families. 

The findings of the study were complemented with the work conducted by [28,29]. These studies were critically reviewed regarding taxonomy, growth habit, altitudinal distribution, types of plants propagation, parts of the plant utilized, and color produced from the species of dye plants, which grow in northern Peru.

**Figure 2:**
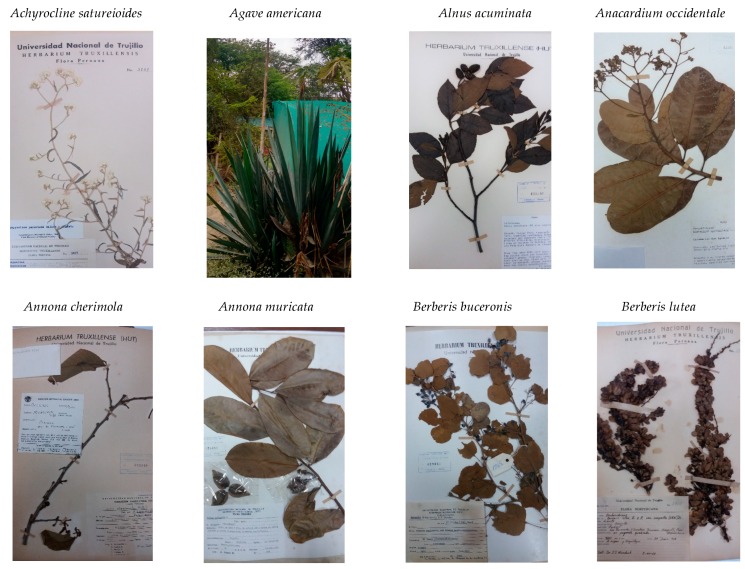

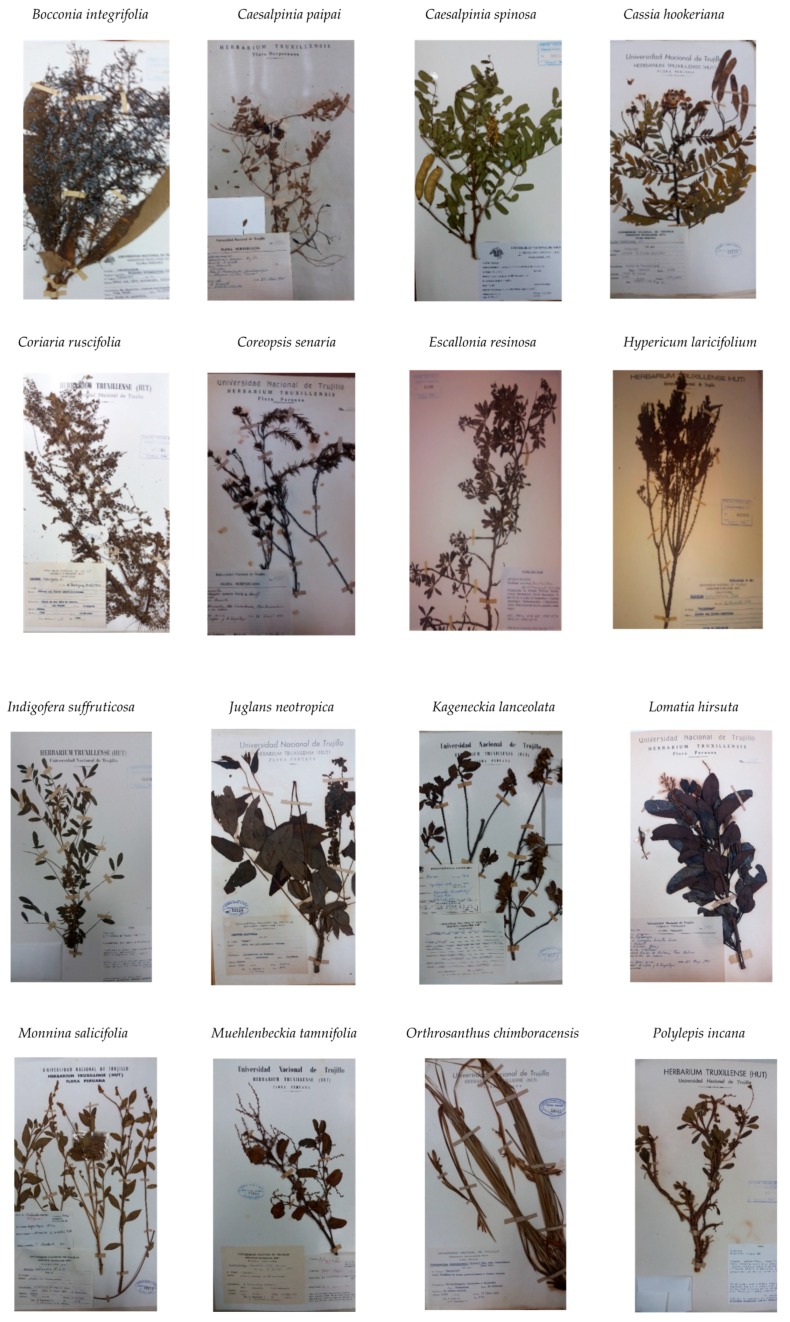

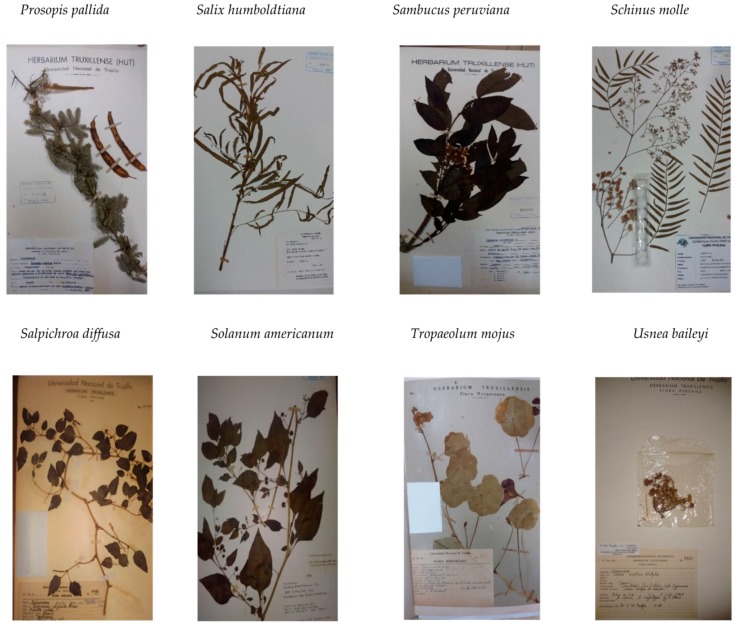
Herbarium specimens of dye plant species registered in the Herbarium Truxillense.

This research also confirms the findings of previous studies in the region including the works of [2,14,27,28]. The specimens collected in Northern Peru, both by the authors of this research and by other botanists, have been registered in the HUT, with their corresponding notes about the properties of the plants in each area, locality, or region (see Figure 2).

Of the 32 dye species identified, five are considered endemic from Peru: *Berberis buceronis*, *Caesalpinia paipai*, *Caesalpinia spinosa*, *Coreopsis senaria*, and *Lomatia hirsuta* [25]. Moreover, with the exception of *Caesalpinia spinosa*, which is endemic to various countries in America, the other four species are endemic to Northern Peru. The other 27 species are widely distributed across the Coast, Highlands, and Amazon regions. It is worth mentioning that *Annona cherimolia* and *Annona muricata* are endemic to both Northern Peru and Southern Ecuador [27]. Species such as *Anacardium occidentale*, *Bocconia integrifolia*, *Agave americana*, *Alnus acuminata*, *Escallonia resinosa*, and *Juglans neotropica* are widely distributed across South and Central America [25].

The results of the inventory record 32 species of dye plants from Northern Peru distributed in 22 families, of which the following stand out due to the number of species: Fabaceae (5), Anacardiaceae (2), Annonaceae (2), Asteraceae (2), Berberidaceae (2), Rosaceae (2), and Solanaceae (2) (see Table 3).

The study also identified many dye plants that are only known by certain Andean communities, which have preserved the old customs or traditions of their use: *Lomatia hirsute*, *Achyrocline satureioides*, *Annona cherimolia*, *Berberis buceronis*, *Coriaria ruscifolia*, *Coreopsis senaria*, *Hypericum laricifolium*, *Monnina salicifolia*, *Orthrosanthus chimboracensis*, and *Salpichroa diffusa*.

We also identified that some plants continue to be used extensively despite the great quantity of synthetic colorants that chemical companies have placed in the market. This is the case for: *Alnusacuminate*, *Bocconia integrifolia*, *Caesalpinia paipai*, *Caesalpinia spinosa*, *Juglans neotropica*, *Kageneckia lanceolata*, and *Polylepis incana*. Finally, some species such as *Bixa orellana*, *Indigofera suffruticosa*, *Sambucus peruviana*, and *Usnea baileyi* constitute a great economic source for several countries [14,27,28]. However, their use has not been industrialized in Peru.

The inventory of northern Peruvian dye plants presented in this article differs in its content from others listed in the bibliography. [28], for example, only offers taxonomic information about some of the species, while [14,27] have inventoried a greater number of species and in addition to taxonomy have included facts concerning the plants’ distribution, parts of the plants used, and the colors produced by some of them. The inventory presented in this article for Northern Peru is more comprehensive, because it is not only based on the number of species listed, but also because of the information provided about the characteristics (taxonomy, growth habit, altitudinal distribution, form of propagation, part of the plant utilized, and the color obtained) of the inventoried species. 

In the botanical world, one cannot generalize that chemicals have a preference to accumulate in a specific plant organ. Therefore, many plants have the principle colorants in their roots, others in the stem, others in the leaves and flowers, and others in the fruit. Of the 32 species analyzed, the principle colorants were found in the stem of 35% of species, 29% in the leaves, 28% in the fruit, 6% in the root, and 3% in the flowers. What can be generalized is that the colorants are deposited according to the order of the percentages mentioned in this research. The natural dyes obtained from the plants are considered an ecological solution to artificial ones. Schmidt-Przewoźna and Brandys (2016) stated that plant extracts contain tannins, flavenoids, saponins, essential oils, mucilage, vitamins, and many other valuable substances, besides being a source of beautiful natural colors [30]. 

## 4. Conclusions

This study aimed to fill the gap that represents the limited systematic documentation and dissemination of the traditional knowledge obtained through the empirical use of many plant species in Northern Peru. The research identified and inventoried 32 species of dye plants that grow in northern Peru, distributed in 22 families, which can be broken down by number of species into the following groups: Fabaceae (5) and Anacardiaceae (2), Annonaceae (2), Asteraceae (2), Berberidaceae (2), Rosaceae (2), and Solanaceae (2). Of the 32 dye species identified, four are considered endemic to Peru: *Berberis buceronis*, *Caesalpinia paipai*, *Coreopsis senaria*, and *Lomatia hirsute*. The study also found that species such as *Bixa orellana*, *Indigofera suffruticosa*, *Sambucus peruviana*, and *Usnea baileyi* have not been commercially exploited in Peru despite the fact that they already constitute a great economic source for several countries. 

This study also compiled the explorations, collections, and records of dye plant species used many generations ago available in the HUT. These explorations gathered facts about the taxonomy, growth habit, altitudinal distribution, methods of propagation, parts of the plant utilized, and color produced from northern Peruvian dye plants. Documenting and preserving traditional knowledge is very important, as it is part of the cultural heritage of indigenous people. 

Finally, as we know, the lack of appropriate documentation on the plant extraction and dyeing techniques is an important factor for the continuous loss of this traditional knowledge. Our future work will focus on investigating and documenting these techniques for their preservation. 

## Figures and Tables

**Table 1:** Botanical exploration routes.

Exploration Number	Places Explored
1	Trujillo, Chiclayo, Piura, Canchaque, Jalca Chingelas, San Ignacio, Cordillera El Condor, Jaen, Chamaya, Olmos, Chiclayo, Trujillo.
2	Trujillo, Chimbote, Pativilca, Laguna Conococha, Chiquian (Bolognesi), Recuay, Huaraz, Carhuaz, Yungay, Caraz, Trujillo.
3	Trujillo, Pativilca, Conococha, Catac, Chavin De Huantar, Huari, Llamellin, Pomabamba, Huascaran, Yungay, Huaraz, Cordillera Negra, Casma, Chimbote, Trujillo.
4	Trujillo, Santa (Chimbote), San Jacinto, Jimbe, Lampanim, Cordillera Negra, Huaylas, Cañon Del Pato, Pampas, Sihuas, Pallasca, Cabana, Corongo, Sihuas, Tayabamba, Buldibuyo, La Paccha, Huamachuco, Shorey, Otuzco, Trujillo.
5	Trujillo, Casa Grande, Cascas, Chuquillanqui, Compin, San Ignacio, Sinsicap, Simbal, Otuzco, Usquil, Coina, Huaranchal, Trujillo.
6	Trujillo, Salpo, Agallpampa, Shorey, Santiago De Chuco, Calipuy, Cachicadan (La Botica), Trujillo.
7	Trujillo, Agallpampa, Shorey, Huamachuco, Pias, Pataz, Parque Nacional Rio Abiseo, Trujillo.
8	Trujillo, Chiclayo, Olmos, Chamaya, Bagua, Pedro Ruiz, Chachapoyas, Rodriguez De Mendoza, Chachapoyas, Leimebamba, Laguna De Los Condores, Jalca Calla Calla, Longotea, Bolivar, Nevado Cajamarquilla, Huayabamba, Valle Del Marañon, Balsas, Gelic, Celendin, Cajamarca, Trujillo.
9	Trujillo, Chiclayo, Olmos, Chamaya, Pomacochas, Rioja, Moyobamba, Tarapoto, Yurimaguas, Bellavista, Juanjui, Tocache, Tingo Maria, Pucallpa, Huanuco, Cerro De Pasco, Lima, Huarmey, Chimbote, Trujillo.
10	Trujillo, Shorey, Huamachuco, Cajabamba, Valle Condebamba, Cajamarca, Porcon, Cumbemayo, Chetilla, Magdalena, Chilete, Tembladera, Trujillo.
11	Trujillo, Chiclayo, Piura, Sullana, Paita, Talara, Mancora, Punta Sal, Tumbes, Parque Nacional Cerros De Amotape, Santuario Nacional Los Manglares, Zarumilla, Trujillo.
12	Trujillo, Chiclayo, Canchaque, Huacabamba, San Antonio, Jalca De Las Huaringas, Ayabaca, Latina, Frontera Con Ecuador, Sullana, Piura, Trujillo.
13	Trujillo, Cascas, Contumaza, Chilete, Cajamarca, Hualgayoc, San Miguel, San Pablo, Chilete, Trujillo.
14	Trujillo, Ascope, San Benito, Guzmango, Cruz Grande Tantarica, Catan, Chilete, Trinidad, Tembladera, Trujillo.
15	Trujillo, Casca,- Contumaza, Cascabamba, Jalca Pozo Kuan, La Asuncion, Cajamarca, Trujillo.
16	Trujillo, Chiclayo, Santa Cruz, Chota, Cutervo, San Andres, Parque Nacional De Cutervo, Chota, Bambamarca, Hualgayoc, Cajamarca, Trujillo.

**Table 2:** Characteristics of dye plant species found in Northern Peru.

Scientific Name	Common Names	Plant Type	Ecology and Distribution in PERU (Meters above Sea Level)	Propagation	Parts Used	Colors Obtained
***Achyrocline satureioides*** **(Lam.) DC—ASTERACE*AE***	árnica, corinilla sacha, wira huayo, huira, vira	Herbaceous plant up to 70 cm in height	From 2500–2800 m Departments: AM, CU, HU, JU, PU, SM.	Seed	The entire plant, [27]	Yellow to green [27]
***Agave americana*** **(L.) a, b—ASPARAGACEAE**	ckara, cabuya, maguey, penca, pinca mara, okcepajpa packpa, pita, cabuya azul, chuchao, pacpa, pappa, maguey mexicano, méxico, kellupancarita.	Succulent with mucronate leaves. Trees up to 10 m tall	Between 500–3200 m.	Bulb	Leaves [26]	Yellow to clear [26,27]
***Alnus acuminata*** **Kunth—BETULACEAE**	aliso, huayau, lambrán, ramras, lambras, ramram, lamra.	Tree 15–30 m in height	Disturbed areas, forest, grassland, riversides, shrublands. From 0–4000 m. Departments: AM, AN, AP, CA, CU, HU, JU, LA, LI, LL, PI, PU	Seed and vegetative	Crushed leaves, bark [26]	Yellow to green. Cinnamon to Brown or from yellow to beige [26,27]
***Anacardium occidentale*** **L. a, b—ANACARDIACEAE**	marañón, caju, casu, cashu, casha, acayocha, cashew, ñucñubaras, ñucñubares	Tree 10 m in height	Disturbed areas. Grows up to 100 m. Departments: CU, HU, JU, LO, PA, SM, UC.	Seed	Fruit (juice)	Yellow
***Annona cherimola*** **Mill. d—ANNONACEAE**	chirimoya	Tree 10 m in height	Grows up to 1500–2200 m Departments: AM, HU, LI.	Seed and vegetative	Bark and fruit	Black
***Annona muricata*** **L. a, b—ANNONACEAE**	guanabana, guanabano, masambo, corosol, cachiman.	Tree between 7–9 m in height	Grows up to 1000 m Department: LO.	Seed and vegetative	Bark and fruit	Black
***Berberis buceronis*** **J.F. Macbr. b—BERBERIDACEAE**	palo amarillo, chulgan	Endemic shrub. Spiny shrub up to 2 m in height	Between 2500–3500 m [28] Department: LL.	Seed	Bark, wood, root, and fruit	Yellow
***Berberis lutea*** **Ruiz & Pav. b—BERBERIDACEAE**	chicchi, ccarahuascassa, espino amarillo, chupite.	Shrub up to 1.5 m tall	Rocky slopes. Grows up to 3800 m. Departments AM, AN, AY, CA, HU, LI, PA, PI, PU, SM.	Bark	Root [27]	Yellow [27]
***Bocconia integrifolia*** **Bonpl. d—PAPAVERACEAE**	ache, picullo, pingullo, pincullo.	Small trees 5 m tall	Rocky slopes. Between 1300–3100 m. Departments: AM, CA, CU, HU, HV, JU, LL, PA, PI, PU.	Seed	Bark, stem, and leaves [26,28]	Yellow and orange
***Caesalpinia paipai*** **Ruiz & Pav.*a*—FABACEAE**	pay-pay, carpe, chara, chorán, pai-pai, tanquis.	Endemic shrub up to 5 m tall	Dry valleys, rocky slopes. Grows up to 800 m. Department: PI.	Seed	Fruit	Black
***Caesalpinia spinosa*** **(Feuillée ex Molina) Kuntze—FABACEAE**	taya, tara, algarroba.	Endemic shrub or tree 3–10 m in height	Lomas, shrublands. From 1500–3100 m. Departments: AM, AN, AR, AY, CA, CU, HU, HV, IC, JU, LI, MD, PI, TA.	Seed	Crushed fruit, fresh fruit, bark, and veins [26,28]	Dark grey to beige. Mordant and fixative. Yellow and green [27]
***Cassia hookeriana*** **Gillies ex Hook. & Arn. b.—FABACEAE**	mutuy	Straight tree 3–4 m in height	From 2700–3800 m. Departments: AM, AP, AY, CA	Seed	Leaves and stem	Yellow and green [27]
***Coriaria ruscifolia*** **L. b.—CORIARIACEAE**	mío-mío, saca-saca	Shrub up to 3 m tall	Disturbed areas, forest, shrublands. From 2500–3800 m. Departments: AM, AP, AY, CA, CU, HU, HV, JU, LL, PU.	Seed	Fruit (juice)	Violet [26]
***Coreopsis senaria*** **S.F. Blake & Sherff—ASTERACEAE**	pull, pagua, pana, panan	Shrub	Grasslands, rocky slopes, shrublands. From 2500–2800 m. Departments: AM, CA, LL.	Seed	Leaves, flower, and stem	Black and yellow
***Escallonia resinosa*** **—Ruiz & Pav. d—ESCALLONIACEAE**	chachas, chacacoma, chachacuma, china-ckenhua, tatás, puca-tirisún, shun, tiriencarnado, siuba	Shrub 2–2.5 m tall. Tree up to 6 m in height	Disturbed areas, forest, shrublands. From 2800–3900 m. Departments: AN, AP, AR, AY, CA, CU, HU, JU, LL.	Seed	Leaves and crushed branches (stem)	Red or flesh-colored and purple. Beige and brown [27]
***Hypericum laricifolium*** **Juss. d—HYPERICACEAE**	chinchaga	Shrub up to 1 m tall	Cloud forest, shrublands. From 2800–3500 m. Departments: AM, AN, CA, HU, LL, PA, PI, SM.	Seed	Stem and leaves	Yellow
***Indigofera suffruticosa*** **Mill. a—FABACEAE**	angaschi, piroañil, añil-añil, llangua, indigo, mutui, mutiucube mutuy.	Shrub up to 2 m tall	Disturbed areas, forests, riversides, and rocky slopes. From 100–1500 m. Departments: AM, AP, AY, CA, CU, HU, JU, LA, LI, LL, LO, PU, SM, TU, UC.	Seed	Leaves	Blue
***Juglans neotropica*** **Diels. d—JUGLANDACEAE**	nogal, nogal del país	Tree between 18 and 30 m in height	Disturbed areas. From 1100–3000 m. Departments: AM, AY, CA, CU, HU, HV, LA, LI, PA.	Seed	Leaves, stem, bark, fruit shell. Fruit juice.	Beige, brown. Yellow to dark brown. Black.
***Kageneckia lanceolata*** **Ruiz & Pav. b—ROSACEAE**	lloque, loque, quisi, uritumicuna	Shrub up to 5 m tall	Disturbed areas, dry valleys, forests, rocky slopes and shrublands. From 2500–3000 m [28] Departments: AM, AN, AP, AY, CA, CU, HU, LI, LL, TA.	Seed	Leaves	Black.
***Lomatia hirsuta*** **(Lam.) Diels. d—PROTEACEAE**	andanga, chotabal, andanca, andagara, gaco, raral, shiapash.	Shrubs up to 3 m in height	Cloud forests, shrub lands. From 2500–3000 m. [28] Departments: CA, LA, PI	Seed	Stem and leaves	Brown.
***Monnina salicifolia*** **Ruiz & Pav. b—POLYGALACEAE**	pichuca, callacón, hachiquís, muchuy, pshuatahuiuac, sambockorota, muchi, ancausa, tutahuiña	Small, woody shrub	Disturbed areas, grasslands, riversides, rocky slopes, semi Deciduous, forests. From 2000–2800 m. Departments: AM, AN, AP, AY, CA, CU, HU, HV, JU, LI, LL, PU.	Seed	Crushed ripe fruit.	Greenish blue.
***Muehlenbeckia tamnifolia*** **(Kunth) Meisn. b—POLYGONACEAE**	*bejuco, bajuco, puma-huascarán*	Shrub or woody plant, climbing plant (vine)	Disturbed areas, elfin forest, rocky slopes and shrublands. From 2200–4500 m. Departments: AM, AN, AP, AR, CA, CU, HU, JU, LII.	Seed	Ripe fruit	Purple
***Orthrosanthus chimboracensis*** **(Kunth) Baker. b—IRIDACEAE**	lirio de jalca, paja purgante, matara, tekel, tuna ocsha, ossapurga	Herbaceous	Disturbed areas, grasslands. From 2500–4000 m. Departments: AM, AN, CA, HU, JU, PI,	Seed	Root	Yellow-orange
***Polylepis incana*** **Kunth. b—ROSACEAE**	quinual, cuña, manzanita, queuña, queños	Tree up to 25 m tall	Dry valleys, forest, shrublands. From 2800–4800 m. Departments.: AN, CU, HU, PA.	Cutting	Leaves or crushed branches	Beige
***Prosopis pallida*** **(Humb. & Bonpl. ex Willd.) Kunth. a—FABACEAE**	algarrobo	Tree 6 to 8 m tall	Desert, rocky slopes, semi-deciduous forests. From 50–1500 m. Departments: AM, AN, AP, AR, CA., HV, IC, LA, LI, LL, PI, TA, TU.	Seed	Stem and leaves	
***Salix humboldtiana*** **Willd.—SALICACEAE**	sauce, huayan, pajaro bobo	Shrub or tree up to 10 m in height	Disturbed, areas, forests. Up to 3000 m. Departments: AM, AR, CU, IC, LA, LI, LO, MD, PI, SM, UC.	Vegetative, cutting	Bark	Brown
***Sambucus peruviana*** **Kunth. a, b—ADOXACEAE**	saúco, arrayán, ccola, kjola (v. aimara), layan, r’ayan, ramrash, yalam	Shrub or tree 4 to 6 m tall	Disturbed areas, forests, rocky slopes, shrub lands. From 2800–3900 m [28]. Departments: AM, CA, CU, HU, LI, PA.	Asexually through cutting	Fruit and leaves	Metallic blue (wine-colored) [28]
***Schinus molle*** **L. a, b—ANACARDIACEAE**	molle, árbol de la vida, cullash, falsa pimienta, huiñam, mulli	Tree 4 to 9 m in height	Rocky slopes. Throughout the Peruvian Andes from 100 to 3200 m. Departments: AN, AR, AY, CA, CU, HU, IC, JU, LI, LL, MO, PA, TA.	Seed	Crushed leaves, mordant bath of leaves, branches, bark, and roots	Yellow to green. Pale yellow
***Salpichroa weberbauerii Dammer*** **Miers. b—SOLANACEAE**	pepenillo, cuytulambo, cuytulum	Shrub up to 2.5 m in height	From 2500–2800 m. Departments: CA, LL, AN, AP, AY, CU, MO	Seed	Fruit	Black
***Solanum americanum*** **Mill.—SOLANACEAE**	hierba mora, ají ccaya-ccaya, cajaya-cajaya, kaya-kaya	Herb up to 50 cm tall	From 10–2500 m. Departments: AM, CA, CU, HU, JU, LA, LI, LO, NID,		Fruit	Blue
***Tropaeolum majus*** **L. a, b—TROPAEOLACEAE**	mastuerzo, aparacay, capuchina mayor, espuela de caballero, tícsau	Herbaceous	Disturbed areas. From 30–2000 m. Departments: AR, CU, LI, LL, TA.	Seed	Plant sap, stems	Yellow
***Usnea baileyi*** **(Stirton) Zahlbr. b—PARMELIACEAE**	líquen, shapra	Surface lichen	From 2500–3500 m [28]	Spores	Entire plant, crushed.	Brown

AM: Amazonas; AN: Ancash; AP: Apurimac; AR: Arequipa; AY: Ayacucho; CA: Cajamarca; CU: Cuzco; HU:Huanuco; HV: Huancavelica; IC: Ica; JU: Junin; LA: Lambayeque; LI: Lima; LL: La Libertad; LO: Loreto; MD: Madre De Dios; MO: Moquegua; PA: Pasco; PI: Piura; PU: Puno; SM: San Martin; TA: Tacna; TU: Tumbes; UC: Ucayali

**Table 3:** Number of dye plant species by family.

Family	Number of Species	Family	Number of Species
FABACEAE	5	ESCALLONIACEAE	1
ASTERACE*AE*	2	HYPERICACEAE	1
ANNONACEAE	2	JUGLANDACEAE	1
BERBERIDACEAE	2	PROTEACEAE	1
ROSACEAE	2	POLYGALACEAE	1
SOLANACEAE	2	POLYGONACEAE	1
ANACARDIACEAE	2	IRIDACEAE	1
ASPARAGACEAE	1	SALICACEAE	1
BETULACEAE	1	ADOXACEAE	1
PAPAVERACEAE	1	TROPAEOLACEAE	1
CORIARIACEAE	1	PARMELIACEAE	1
